# Supplementary material for: Spatiotemporal dynamics of moiré excitons in van der Waals heterostructures
Source: Nat Commun. 2025 Sep 29;16:8557. doi: 10.1038/s41467-025-64285-2 (PMC12480941; doi:10.1038/s41467-025-64285-2)
Supplement: Supplementary file 1 — Supplementary Information [file 41467_2025_64285_MOESM1_ESM.pdf]

# Spatiotemporal dynamics of moiré excitons in Van der Waals heterostructures
## Supplementary Information

Giuseppe Meneghini,[1,*] Samuel Brem,[1] and Ermin Malic[1]

[1] *Department of Physics, Philipps University of Marburg, 35037 Marburg, Germany*

### THEORETICAL APPROACH

In this section, we present a detailed derivation of the equations introduced in the main text. We begin by defining the system Hamiltonian and the necessary change of basis to arrive at the moiré exciton formalism. Subsequently, we introduce the Wigner representation to derive the Boltzmann transport equation. Finally, we provide additional details on the Monte Carlo implementation and the evaluation of the scattering rate integrals.

#### Moiré Exciton Hamiltonian

We start by defining the excitonic Hamiltonian in second quantization for a TMD bilayer in the low excitation regime, including the exciton-phonon interaction and yielding[1,2]

$$H = \sum_{\mu\mathbf{Q}} \mathcal{E}^{\mu}_{\mathbf{Q}} X^{\mu\dagger}_{\mathbf{Q}} X^{\mu}_{\mathbf{Q}} + \sum_{j\mathbf{Q}\mathbf{q}\mu\nu} \tilde{\mathcal{D}}^{\mu\nu}_{j\mathbf{q}\mathbf{Q}} X^{\nu\dagger}_{\mathbf{Q}+\mathbf{q}} X^{\mu}_{\mathbf{Q}} b_{j,\mathbf{q}} + h.c. \tag{1}$$

Here, we have introduced exciton creation (annihilation) operators $X^{\mu(\dagger)}_{\mathbf{Q}}$, creating (annihilating) an exciton in the state $\mu$ at the center of mass momentum $\mathbf{Q}$. Using a valley-local representation, we can split the sum over $\mathbf{Q} \in BZ$ in $\mathbf{Q} + \xi_e - \xi_h$ (with the new $\mathbf{Q}$ in an interval around each high-symmetry point $\xi$) defining the super index $\mu = (n^{\mu}, \xi^{\mu}_e, \xi^{\mu}_h, l^{\mu}_e, l^{\mu}_h)$, with $n$ describing the series of Rydberg-like states determining the relative electron-hole motion, and $l$ denoting the electron/hole layer. We have, furthermore, introduced the exciton energy $\mathcal{E}^{\mu}_{\mathbf{Q}} = \hbar^2\mathbf{Q}^2/(2M_{\mu}) + E^g_{\mu} + E^b_{\mu}$ with the mass $M^{\mu} = m^{\mu}_e + m^{\mu}_h$ ($m_{e/h}$ electron/hole mass), $E^g_{\mu}$ corresponding to the energy gap between the valence and the conduction band, and $E^b_{\mu}$ denoting the exciton binding energy, obtained from solving the Wannier equation. The phonon operators $b^{(\dagger)}_{j,\mathbf{q}}$ create (annihilate) a phonon with the momentum $\mathbf{q}$ and the compound mode index $j = (\kappa_j, \xi^{ph}_j, l^{ph}_j)$, where $\kappa$ is the phonon mode (acoustic or optical modes), while $\xi$ and $l$ denote the phonon valley and layer, respectively. Moreover, we have introduced the exciton-phonon coupling element $\tilde{\mathcal{D}}^{\nu\mu}_{j\mathbf{q}\mathbf{Q}}$ reading

$$\begin{aligned} \tilde{\mathcal{D}}^{\mu\nu}_{j,\mathbf{q},\mathbf{Q}} = {} & D^{\xi^e_{\mu}\xi^e_{\nu}c}_{j,\mathbf{q}} \delta_{\xi^h_{\mu}\xi^h_{\nu}} \delta_{\xi^e_{\nu}-\xi^e_{\mu},\xi^{ph}_j} \delta_{l^e_{\nu},l^{ph}_j} \delta_{l^e_{\nu},l^e_{\mu}} \mathcal{F}^{\mu\nu}\left(\frac{m^{\nu}_h}{M^{\nu}}\left[\mathbf{q} + s_{\mu\nu}\mathbf{Q}\right]\right) + \\ & - D^{\xi^h_{\mu}\xi^h_{\nu}v}_{j,\mathbf{q}} \delta_{\xi^e_{\mu}\xi^e_{\nu}} \delta_{\xi^h_{\nu}-\xi^h_{\mu},\xi^{ph}_j} \delta_{l^h_{\nu},l^{ph}_j} \delta_{l^h_{\nu},l^h_{\mu}} \mathcal{F}^{\mu\nu}\left(-\frac{m^{\nu}_e}{M^{\nu}}\left[\mathbf{q} + s_{\mu\nu}\mathbf{Q}\right]\right). \end{aligned} \tag{2}$$

Here, we use the subscript $ph$ to label phonon quantum numbers. The terms $\delta_{\xi^{e/h}_{\mu}\xi^{e/h}_{\nu}} \delta_{\xi^{h/e}_{\nu}-\xi^{h/e}_{\mu},\xi^{ph}_j}$ fix the momentum conservation of each scattering process with respect to the total phonon momentum $\tilde{\mathbf{q}} = \xi^{ph}_j + \mathbf{q}$. Here, $\mathcal{F}^{\mu\nu}(\mathbf{q}) = \sum_{\mathbf{k}} \psi^{\mu *}(\mathbf{k})\psi^{\nu}(\mathbf{k}+\mathbf{q})$ are the excitonic form factors obtained from the excitonic eigenfunction $\psi^{\mu}(\mathbf{k})$ derived from the Wannier equation, and $s_{\mu\nu} = 1 - M_{\nu}/M_{\mu}$ is the mass imbalance. Furthermore, $D^{\xi^{\lambda}_m\xi^{\lambda}_n\lambda}_{j,\mathbf{q}}$ denotes the electron/hole-phonon coupling element for TMD monolayers, taken from first-principle

calculations[3], yielding

$$D^{\xi^\lambda_m\xi^\lambda_n\lambda}_{j,\mathbf{q}} \approx \sqrt{\frac{\hbar}{2\rho_{l^{ph}_j} A\Omega_{j\mathbf{q}}}}\tilde{D}^{\xi^\lambda_m\xi^\lambda_n\lambda}_{j,\mathbf{q}}$$

$$\text{with}\quad \tilde{D}^{\xi^\lambda_m\xi^\lambda_n\lambda}_{j,\mathbf{q}} = \begin{cases}\tilde{D}^\lambda_\xi \mathbf{q} & \text{if } \xi^\lambda_m = \xi^\lambda_n = \xi \quad \text{and } \kappa_j = TA, LA \\ \tilde{D}^\lambda_{\xi^\lambda_m\xi^\lambda_n} & \text{else}\end{cases} \tag{3}$$

$$\text{and}\quad \Omega_{j\mathbf{q}} = \begin{cases} v_j\mathbf{q} & \text{if } \kappa_j = TA, LA \\ \Omega_{_j} & \text{else.}\end{cases}$$

Here, $\lambda = c, v$ corresponds to the electronic band index, $TA, LA$ to the acoustic transversal and longitudinal phonon modes, and with "else" we refer to optical modes and intervalley contributions. Furthermore, $A$ denotes the area of the system, $\rho_{l^{ph}_j}$ the surface mass density in the specific phonon layer $l^{ph}_j$, and $v_j$ the sound velocity in the TMD layer. We have used a zeroth order Taylor expansion in vicinity of high-symmetry points to model phonon energy and obtain a material-specific description, using a linear dispersion (Debye approximation) for acoustic modes around the $\Gamma$ point, while for optical and short-wavelength acoustic phonons ($\xi \neq \Gamma$) we use a constant energy (Einstein approximation). To obtain the previous electron-phonon coupling, a deformation potential approximation has been applied, where the full electron-phonon matrix element has been approximated with a zeroth/first order Taylor expansion.

In our study, we focus on the lowest state, which is the interlayer KK 1s exciton for the considered $WSe_2$-$MoSe_2$ heterostructure. As shown in DFT calculations[4], electronic orbitals are localized around the inner metal atoms in proximity of the K point, and thus the hybridization (delocalization of electron/hole accross the layers) is relatively weak.

We know from first-principle calculations that the electron/hole tunneling is negligible around the K valley in the considered TMD heterostructure[5]. In addition, the largest contribution to the electronic states at the K point arises from d-orbitals at the metal atoms, which are protected from the environment by the surrounding chalcogen atoms. To model the twist-angle dependent energy modulation, we therefore assume that the electronic Hamiltonian contains effective electrostatic potentials $\hat{W}_l$ (with $l = 0, 1$ for the two layers) created by the lattices of the two vertically stacked layers yielding

$$H = \sum_{ll'\mathbf{k}\mathbf{k}'} \langle l\mathbf{k}|\, \hat{T} + \hat{W}_0 + \hat{W}_1 \,|l'\mathbf{k}'\rangle\, a^\dagger_{l\mathbf{k}} a_{l'\mathbf{k}'} = \sum_{l\mathbf{k}} \varepsilon_{l\mathbf{k}} a^\dagger_{l\mathbf{k}} a_{l\mathbf{k}} + \sum_{l\mathbf{k}\mathbf{q}} M_{l\mathbf{k}}(\mathbf{q}) a^\dagger_{l\mathbf{k}+\mathbf{q}} a_{l\mathbf{k}} \tag{4}$$

where $\langle \mathbf{r}|l\mathbf{k}\rangle = \psi^l_{\mathbf{k}}(\mathbf{r}) = \frac{1}{\sqrt{N}}\sum_{\mathbf{R}_l} e^{i(\mathbf{k}+\mathbf{K}_l)\cdot\mathbf{R}_l}\phi_l(\mathbf{r}-\mathbf{R}_l)$ are the monolayer eigenstates at the K point composed of the orbitals $\phi_l$ at metal-atom sites $\mathbf{R}_l$. Furthermore, we have introduced the kinetic energy $\hat{T} = \hat{K}_0 + \hat{K}_1$, the monolayer band energies $\varepsilon_{l\mathbf{k}} = \langle l\mathbf{k}|\, \hat{K}_l + \hat{W}_l \,|l\mathbf{k}\rangle$, and the moiré potential $M_{l\mathbf{k}}(\mathbf{q}) = \langle l\mathbf{k}|\, \hat{W}_{1-l} \,|l\mathbf{k}\rangle$. Expanding the moiré term in atomic contributions $W_l = \sum_{\mathbf{R}_l} w_l(\mathbf{r}-\mathbf{R}_l)$ we can rewrite it as $M_{l\mathbf{k}}(\mathbf{q}) = \sum_{\mathbf{G}_l\mathbf{G}_{1-l}} m_l(\mathbf{G}_{1-l}) e^{i(\mathbf{G}_l+\mathbf{G}_{1-l})\cdot\mathbf{D}_l/2}\delta_{\mathbf{q},\mathbf{G}_{1-l}-\mathbf{G}_l}$ with the reciprocal lattice vectors $G_l$ and the atomic interaction energy $m_l(\mathbf{q}) = 1/A_{UC}\int dz \tilde{\rho}_l(-\mathbf{q}, z)\tilde{w}_l(\mathbf{q}, z)$ using $\rho_l(\mathbf{r}) = |\phi_l(\mathbf{r})|^2$ and the in-plane Fourier transform $\tilde{f}(\mathbf{q}, z) = \int d\mathbf{r}_\parallel f(\mathbf{r}) e^{i\mathbf{q}\mathbf{r}_\parallel}$. Here, $A_{UC}$ is the area of the unit cell and the quantity $\mathbf{D}_l = \mathbf{R}^0_l - \mathbf{R}^0_{1-l}$ is defined as the spatial displacement of the two lattice origins. Now we exploit the fact that the atomic potentials are smooth functions, so that we can restrict the sum over reciprocal lattice vectors to the first shell. Terms with $\mathbf{G}_l = \mathbf{G}_{1-l} = 0$ lead to stacking- and space-independent band shifts. Furthermore, we can decompose the atomic potential into parts stemming from metal- and chalcogen atoms and exploit the $C_3$ symmetry of orbitals/atomic potentials in a hexagonal lattice, which finally yields

$$V_M = \sum_{l\mathbf{k}\mathbf{q}} V_{l\mathbf{q}} a^\dagger_{l\mathbf{k}+\mathbf{q}} a_{l\mathbf{k}} + h.c. \tag{5}$$

$$V_{l\mathbf{q}} = v_l \sum_{n=0}^{2} e^{iC^n_3(\mathbf{G}_l+\mathbf{G}_{1-l})\cdot\mathbf{D}_l/2}\delta_{\mathbf{q},C^n_3(\mathbf{G}_{1-l}-\mathbf{G}_l)} \tag{6}$$

with $v_l = \gamma^l_1 + \gamma^l_2 e^{2\pi\sigma_{1-l}/3}$, where $\sigma_l = 1/-1$ for R-type/H-type stacking configurations. Hence, the potential is fully parametrized by the two numbers $\gamma^l_1$ and $\gamma^l_2$, characterizing the interaction energy of the K-point

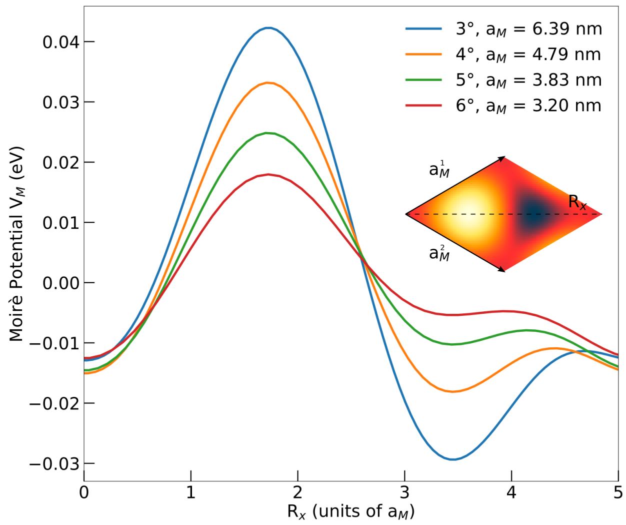


Fig. S1. Real space moiré potential for the different twist angles studied in this work. The potential is plotted in units of the corresponding moiré lattice constant $a_M$

orbitals with metal- and chalcogen-atoms of the other layer, respectively. These can be directly obtained from first-principle calculations by comparing the band energies for different high-symmetry stackings, i.e., by fixing the twist angle to 0° and considering $\mathbf{G}_0 \simeq \mathbf{G}_1$, the potential depends only on the lateral displacements $\mathbf{D}_l$. As a result, using the energy levels from $R_{hh}, R_{Mh}$ and $R_{Xh}$ allows us to unambiguously determine $\gamma^l_{1/2}$[5]. Figure S1 shows the moiré potential for the different twist angles studied in the main text.

Moving to the exciton representation and considering only the interlayer contribution, we obtain

$$V_M = \sum_{\mathbf{Q}\tilde{\mathbf{g}}\mathbf{g}} \mathcal{M}_{\tilde{\mathbf{g}}\mathbf{g}} X^\dagger_{\mathbf{Q}\tilde{\mathbf{g}}} X_{\mathbf{Q}\mathbf{g}} \tag{7}$$

where the exciton quantum number $\mu$ is fixed to be the exciton ground state and thus omitted in the following. Here, we moved to a folded representation of exciton operators exploiting the new symmetry of the potential, with $\mathbf{Q} \in 1^{st}$mBZ and $\mathbf{g} = s_1\mathbf{G}^{\mathbf{M}}_{\mathbf{1}} + s_2\mathbf{G}^{\mathbf{M}}_{\mathbf{2}}$ with $\mathbf{G}^{\mathbf{M}}_{\mathbf{1/2}}$ as the reciprocal moiré lattice vector and $s_{1/2}$ as integers (where we use MBZ to refer to the moiré Brillouin zone accounting for the new periodicity arising from the moiré potential). We define the moiré matrix elements as follows

$$\begin{aligned} \mathcal{M}_{\tilde{\mathbf{g}}\mathbf{g}} = \Theta \left(\delta_{s_1,\tilde{s_1}+(-1)^{l_e}}\delta_{s_2,\tilde{s_2}} + \delta_{s_1,\tilde{s_1}}\delta_{s_2,\tilde{s_2}+(-1)^{l_e}} + \delta_{s_1,\tilde{s_1}+(-1)^{l_e}}\delta_{s_2,\tilde{s_2}+(-1)^{l_e}}\right) + \\ + \Theta^* \left(\delta_{s_1,\tilde{s_1}-(-1)^{l_e}}\delta_{s_2,\tilde{s_2}} + \delta_{s_1,\tilde{s_1}}\delta_{s_2,\tilde{s_2}-(-1)^{l_e}} + \delta_{s_1,\tilde{s_1}-(-1)^{l_e}}\delta_{s_2,\tilde{s_2}-(-1)^{l_e}}\right) \end{aligned} \tag{8}$$

where $\Theta = v^c_{l_e}\mathcal{F}(\frac{m_h}{M}\mathbf{g_0}) - v^{v*}_{l_h}\mathcal{F}(\frac{m_e}{M}\mathbf{g_0})$ with $v^{c/v}_{l_{e/h}} = \gamma^{c/v}_1 + \gamma^{c/v}_2 e^{2\pi i/3}$ as the effective atomic potentials for the conduction and the valence bands in the neighboring layer. The parameters $\gamma^{c/v}_{1/2}$ are defined from the $\gamma^l_{1/2}$ by fixing the layer index to be the conduction/valence band one, and $\mathbf{g_n} = \mathrm{C}_3^{n-1}(\mathbf{G^1_1} - \mathbf{G^0_1})$, where $G^l_m$ refers to the $m = 1, 2$ reciprocal lattice vector for layer $l = 0, 1$. We include the moiré potential into the free exciton Hamiltonian, reading in the folded representation[5] $H_M = \sum_{\mathbf{Qg}} \mathcal{E}_{\mathbf{Qg}} X^\dagger_{\mathbf{Qg}} X_{\mathbf{Qg}} + \sum_{\mathbf{Q}\tilde{\mathbf{g}}\mathbf{g}} \mathcal{M}_{\tilde{\mathbf{g}}\mathbf{g}} X^\dagger_{\mathbf{Q}\tilde{\mathbf{g}}} X_{\mathbf{Qg}}$. This Hamiltonian is diagonal for moiré excitons, i.e. $Y^{\eta(\dagger)}_{\mathbf{Q}} = \sum_{\mathbf{g}} \omega^{\eta(*)}_{\mathbf{g}}(\mathbf{Q}) X^{(\dagger)}_{\mathbf{Qg}}$, when the momentum-mixing coefficients $\omega^{\eta(*)}_{\mathbf{g}}(\mathbf{Q})$ fulfill the eigenvalue problem

$$\mathcal{E}_{\mathbf{Qg}}\omega^\eta_{\mathbf{g}}(\mathbf{Q}) + \sum_{\tilde{\mathbf{g}}} \mathcal{M}_{\tilde{\mathbf{g}}\mathbf{g}}\omega^\eta_{\tilde{\mathbf{g}}}(\mathbf{Q}) = E^\eta_{\mathbf{Q}}\omega^\eta_{\mathbf{g}}(\mathbf{Q}). \tag{9}$$

giving rise two a new set of energy mini-bands $E^\eta_{\mathbf{Q}}$. Using these states to perform a change of basis in the

full Hamiltonian in Eq. (1) leads us to the final Hamilton operator

$$\tilde{H} = \sum_{\eta} E^{\eta}_{\mathbf{Q}} Y^{\eta\dagger}_{\mathbf{Q}} Y^{\eta}_{\mathbf{Q}} + \sum_{j\mathbf{q}} \Omega_{\mathbf{jq}} b^{\dagger}_{j\mathbf{q}} b_{j\mathbf{q}} + \sum_{\substack{\eta\xi j \\ \mathbf{QQ'g}}} \tilde{\mathcal{D}}^{\eta\xi j}_{\mathbf{g}}(\mathbf{Q},\mathbf{Q'}) Y^{\xi\dagger}_{\mathbf{Q'}} Y^{\eta}_{\mathbf{Q}} (b^{j}_{\mathbf{Q'-Q+g}} + b^{j\dagger}_{\mathbf{Q'-Q-g}}) \tag{10}$$

with $\Omega_{j\mathbf{q}}$ as the energy of the phonon mode $j$. The moiré exciton-phonon coupling elements are defined as

$$\tilde{\mathcal{D}}^{\eta\xi j}_{\mathbf{g}}(\mathbf{Q},\mathbf{Q'}) = \sum_{\mathbf{g'}} \tilde{\mathcal{D}}_{j\mathbf{Q'-Q+g},\mathbf{Q}} \omega^{\eta *}_{\mathbf{g'}}(\mathbf{Q}) \omega^{\xi}_{\mathbf{g-g'}}(\mathbf{Q'}). \tag{11}$$

These are expressed in terms of exciton-phonon coupling elements $\tilde{\mathcal{D}}_{j\mathbf{Q'-Q+g},\mathbf{Q}}$ defined in Eq. (2).

## Boltzmann transport equation for moiré excitons

To study the spatiotemporal exciton dynamics, we solve the Heisenberg equation of motion for off-diagonal terms of the moiré exciton density matrix, i.e. $\rho^{\eta}_{\mathbf{QQ'}} = \left\langle Y^{\eta\dagger}_{\mathbf{Q}} Y^{\eta}_{\mathbf{Q'}} \right\rangle$. In the following, extending the approach introduced by Hess and Kuhn[6–9], we focus on one band and one phonon mode (we will omit the state index $\eta$ and the phonon mode index $j$) for simplicity, but the result can be easily generalized to the case of multiple bands and phonon modes. From the Heisenberg equation of motion for $\rho^{\eta}_{\mathbf{QQ'}}$ we obtain the two coupled equations

$$(i\hbar\partial_t + E_{\mathbf{Q}} - E_{\mathbf{Q'}})\,\rho_{\mathbf{QQ'}} = \sum_{\xi\mathbf{Pqg}} \tilde{\mathcal{D}}_{\mathbf{g}}(\mathbf{Q'},\mathbf{P}) \left(S_{\mathbf{QPq+g}} + S^{*}_{\mathbf{QP\text{-}q\text{-}g}}\right) - \tilde{\mathcal{D}}^{*}_{\mathbf{g}}(\mathbf{P},\mathbf{Q}) \left(S_{\mathbf{PQ'q+g}} + S^{*}_{\mathbf{PQ'\text{-}q\text{-}g}}\right) \tag{12}$$

$$\begin{aligned} \partial_t S_{\mathbf{QPq+g}} = &-\frac{i}{\hbar}\left(E_{\mathbf{Q'}} - E_{\mathbf{Q}} - \Omega_{\mathbf{q+g}} + i\Gamma_{\mathbf{Q}} + i\Gamma_{\mathbf{Q'}}\right) S_{\mathbf{QPq+g}} \\ &-\frac{i}{\hbar}\sum_{\mathbf{P}}\left[\tilde{\mathcal{D}}^{*}_{\mathbf{g}}(\mathbf{P},\mathbf{Q'})\rho_{\mathbf{QP}}(n_{\mathbf{q+g}}+1) - \tilde{\mathcal{D}}^{*}_{\mathbf{g}}(\mathbf{Q},\mathbf{P})\rho_{\mathbf{PQ'}} n_{\mathbf{q+g}}\right] \end{aligned} \tag{13}$$

where we introduced $S_{\mathbf{QPq}} = \left\langle Y^{\dagger}_{\mathbf{Q}} Y_{\mathbf{P}} b_{\mathbf{q}} \right\rangle$, the Bose-Einstein phonon occupation $n_{\mathbf{q}}$, and neglected quadratic terms in $\rho$ (low density limit) and included collisional broadening corrections stemming from the third order of the cluster expansion ($\Gamma_{\mathbf{Q}} + \Gamma_{\mathbf{Q'}}$). If we now restrict to elements close to the diagonal in the density matrix, i.e. $|\mathbf{l}| << mBZ$ (translated in real space, this means that we consider envelope varying slowly with respect to the unit cell of the moiré lattice), we can define $\rho_{\mathbf{Q}}(\mathbf{l}) = \left\langle Y^{\dagger}_{\mathbf{Q}} Y_{\mathbf{Q\text{-}l}} \right\rangle$, arriving at

$$\begin{aligned} \frac{d}{dt}\rho_{\mathbf{Q}}(\mathbf{l}) = &\frac{i}{\hbar}(E_{\mathbf{Q-l}} - E_{\mathbf{Q}})\rho_{\mathbf{Q}}(\mathbf{l}) \\ &-\frac{i}{\hbar}\sum_{\mathbf{gPq}} |D_{\mathbf{g}}(\mathbf{Q},\mathbf{P})|^2 \Bigg[ W^{\mathbf{QP}}_{\mathbf{Q-P+g}}\rho_{\mathbf{Q}}(-\mathbf{q})\delta_{-\mathbf{q},\mathbf{l}} + W^{\mathbf{PQ}*}_{\mathbf{P-Q-g}}\rho_{\mathbf{Q+q}}(\mathbf{l+q})\delta_{\mathbf{q},0} \\ &- W^{\mathbf{QP}*}_{\mathbf{Q-P+g}}\rho_{\mathbf{P-l+q}}(\mathbf{q})\delta_{\mathbf{q},\mathbf{l}} - W^{\mathbf{PQ}}_{\mathbf{P-Q-g}}\rho_{\mathbf{Q}}(\mathbf{l-q})\delta_{\mathbf{q},0}\Bigg] \end{aligned} \tag{14}$$

following from a second order Born-Markov approximation, with definition of $W^{\mathbf{mn}}_{\mathbf{q}} = n_{\mathbf{q}} \mathrm{L}^{\mathbf{mn}}_{\mathbf{q}} - (n_{\text{-}\mathbf{q}}+1)\mathrm{L}^{\mathbf{nm}*}_{\text{-}\mathbf{q}}$ and $\mathrm{L}^{\mathbf{mn}}_{\mathbf{q}} = (E_{\mathbf{m}} - E_{\mathbf{n}} + \Omega_{\mathbf{q}} - i(\Gamma_{\mathbf{m}} + \Gamma_{\mathbf{n}}))^{-1}$. Here we used $|\mathbf{l}| << mBZ$ to approximate $D_{\mathbf{g}}(\mathbf{Q-l},\mathbf{P-l}) \simeq D_{\mathbf{g}}(\mathbf{Q},\mathbf{P})$ and $E_{\mathbf{Q-l}} - E_{\mathbf{Q'-l}} \simeq= E_{\mathbf{Q}} - E_{\mathbf{Q'}}$ in $W^{\mathbf{Q-l},\mathbf{Q'-l}}_{\mathbf{q}} \simeq W^{\mathbf{Q},\mathbf{Q'}}_{\mathbf{q}}$. By Fourier transforming and by introducing the Wigner representation $f_{\mathbf{Q}}(\mathbf{r}) = \sum_{\mathbf{l}\in mBZ} e^{i\mathbf{l}\cdot\mathbf{r}}\rho_{\mathbf{Q}}(\mathbf{l})$, we arrive at

$$\frac{d}{dt}f_{\mathbf{Q}}(\mathbf{r}) = -\mathbf{v_Q}\nabla_{\mathbf{r}} f_{\mathbf{Q}}(\mathbf{r}) + \frac{2}{\hbar}\sum_{\mathbf{gP}} |D_{\mathbf{g}}(\mathbf{Q},\mathbf{P})|^2 \left[\mathrm{Im}\left(W^{\mathbf{QP}}_{\mathbf{Q-P+g}}\right) f_{\mathbf{P}}(\mathbf{r}) - \mathrm{Im}\left(W^{\mathbf{PQ}}_{\mathbf{P-Q-g}}\right) f_{\mathbf{Q}}(\mathbf{r})\right] \tag{15}$$

where we indicate the imaginary part as Im, and we have used that $i/\hbar\sum_{\mathbf{l}} e^{i\mathbf{l}\cdot\mathbf{r}}(E_{\mathbf{Q-l}} - E_{\mathbf{Q}})\rho_{\mathbf{Q},\mathbf{Q\text{-}l}} \simeq i/\hbar\sum_{\mathbf{l}} e^{i\mathbf{l}\cdot\mathbf{r}}\mathbf{l}\cdot\nabla_{\mathbf{Q}} E_{\mathbf{Q}}\rho_{\mathbf{Q},\mathbf{Q\text{-}l}} = -1/\hbar\nabla_{\mathbf{Q}} E_{\mathbf{Q}}\nabla_{\mathbf{r}} f_{\mathbf{Q}}(\mathbf{r}) = -\mathbf{v_Q}\nabla_{\mathbf{r}} f_{\mathbf{Q}}(\mathbf{r})$ with $\mathbf{v_Q}$ group velocity. Noticing that

$\mathrm{Im}\left(W_{\mathbf{q}}^{\mathbf{QP}}\right) = n_{\mathbf{q}}\mathrm{Im}(\mathrm{L}_{\mathbf{q}}^{\mathbf{mn}}) - (n_{\mathbf{-q}}+1)\mathrm{Im}(\mathrm{L}_{\mathbf{-q}}^{\mathbf{nm}*})$ and that $\mathrm{Im}(\mathrm{L}_{\mathbf{q}}^{\mathbf{mn}}) = \pi\mathcal{L}(E_{\mathbf{m}} - E_{\mathbf{n}} + \Omega_{\mathbf{q}})$ with $\mathcal{L}$ Lorentzian function, we can define $\tilde{W}_{\mathbf{QQ}'} = \frac{2\pi}{\hbar}\sum_{\mathbf{g}} |D_{\mathbf{g}}(\mathbf{Q},\mathbf{Q}')|^2 W_{\mathbf{Q}-\mathbf{Q}'+\mathbf{g}}^{\mathbf{QQ}'}$. With this we arrive finally at the Boltzmann transport equation for moiré excitons (reintroducing the dependence on the band indices)

$$\frac{d}{dt}f_{\mathbf{Q}}^{\eta}(\mathbf{r}) = -\mathbf{v}_{\mathbf{Q}}^{\eta}\nabla_{\mathbf{r}}f_{\mathbf{Q}}^{\eta}(\mathbf{r}) + \sum_{\xi\mathbf{P}}\left[\tilde{W}_{\mathbf{PQ}}^{\xi\eta}f_{\mathbf{P}}^{\xi}(\mathbf{r}) - \tilde{W}_{\mathbf{QP}}^{\eta\xi}f_{\mathbf{Q}}^{\eta}(\mathbf{r})\right]. \tag{16}$$

Here, the time evolution of the Wigner function for moiré excitons $\eta$ at momentum $\mathbf{Q}$ and real space position $\mathbf{r}$ is governed by a drift term, which is dependent on the group velocity and the spatial gradient of the Wigner function, and a collision term, which describes phonon-mediated exciton transitions between initial and final states. To extract the diffusion coefficient, we consider the time evolution of an initially Gaussian exciton spatial profile. In the stationary regime, after excitons have reached a steady energy-momentum distribution, the real-space dynamics follows Fick's law[9,10]. In this regime, the variance of the spatial distribution grows linearly with time, allowing the diffusion coefficient to be estimated as

$$D = \frac{1}{4}\partial_t\sigma_t^2. \tag{17}$$

**Diffusion coefficient**

To perform a quantitative comparison with both the Boltzmann-distributed case and the purely parabolic regime, we derive an analytical expression for the diffusion coefficient extending the approach introduced by Hess and Kuhn[6–9], by assuming a stationary distribution close to equilibrium and by applying the relaxation time approximation[6]. Focusing on the collisional term in Eq. 16, we write

$$\left.\frac{d}{dt}f_{\mathbf{Q}}^{\eta}(\mathbf{r})\right|_{col} = \sum_{\xi\mathbf{P}}\left[\tilde{W}_{\mathbf{PQ}}^{\xi\eta}\left(f_{\mathbf{P}}^{\xi 0}(\mathbf{r}) + \delta f_{\mathbf{P}}^{\xi}(\mathbf{r})\right) - \tilde{W}_{\mathbf{QP}}^{\eta\xi}\left(f_{\mathbf{Q}}^{\eta 0}(\mathbf{r}) + \delta f_{\mathbf{Q}}^{\eta}(\mathbf{r})\right)\right] \tag{18}$$

$$= -\delta f_{\mathbf{Q}}^{\eta}(\mathbf{r})\sum_{\xi\mathbf{P}}\tilde{W}_{\mathbf{PQ}}^{\xi\eta} = -\Gamma_{\mathbf{Q}}^{\eta}\delta f_{\mathbf{Q}}^{\eta}(\mathbf{r}) \tag{19}$$

where we assume that the system is close to a local equilibrium, so that the distribution $f_{\mathbf{Q}}^{\eta}(\mathbf{r})$ can be decomposed into its equilibrium component $f_{\mathbf{Q}}^{\eta 0}(\mathbf{r})$ and a small deviation $\delta f_{\mathbf{Q}}^{\eta}(\mathbf{r})$. Here, we have introduced the total out-scattering rate $\Gamma_{\mathbf{Q}}^{\eta} = \sum_{\xi\mathbf{P}}\tilde{W}_{\mathbf{PQ}}^{\xi\eta}$. Inverting the above expression yields

$$\delta f_{\mathbf{Q}}^{\eta}(\mathbf{r}) = -\tau_{\mathbf{Q}}^{\eta}\left[\frac{d}{dt} + \mathbf{v}_{\mathbf{Q}}^{\eta}\nabla_{\mathbf{r}}\right]f_{\mathbf{k}}^{\eta 0}(\mathbf{r}) \tag{20}$$

with scattering time $\tau_{\mathbf{Q}}^{\eta} = 1/\Gamma_{\mathbf{Q}}^{\eta}$. We now consider the relation between the intraband current and the particle density, given by $j(\mathbf{r}) = -\mathbf{D}\cdot\nabla_{\mathbf{r}}N(\mathbf{r})$[6], where $\mathbf{D}$ is the diffusion tensor and $N(\mathbf{r}) = \sum_{\mathbf{k},\eta}f_{\mathbf{k}}^{\eta}(\mathbf{r})$. The current can also be expressed as

$$j(\mathbf{r}) = 1/A\sum_{\mathbf{k}\eta}\mathbf{v}_{\mathbf{k}}^{\eta}f_{\mathbf{k}}^{\eta}(\mathbf{r}) \tag{21}$$

where $A$ is the area of the system[7–9]. Assuming the distribution is close to equilibrium, we substitute $f_{\mathbf{k}}^{\eta}(\mathbf{r}) = f^{\eta 0}\mathbf{k}(\mathbf{r}) + \delta f^{\eta}\mathbf{k}(\mathbf{r})$ and obtain

$$j(\mathbf{r},t) = \frac{1}{A}\sum_{\mathbf{k}\eta}\mathbf{v}_{\mathbf{k}}^{\eta}\left(f_{\mathbf{k}}^{\eta 0}(\mathbf{r}) + \delta f_{\mathbf{k}}(\mathbf{r})\right) = \frac{1}{A}\sum_{\mathbf{k}\eta}\mathbf{v}_{\mathbf{k}}^{\eta}\left[\frac{d}{dt} + \mathbf{v}_{\mathbf{Q}}^{\eta}\nabla_{\mathbf{r}}\right]f_{\mathbf{k}}^{\eta 0}(\mathbf{r}) \tag{22}$$

where, in the last step, we used the expression for $\delta f_{\mathbf{k}}^{\eta}(\mathbf{r})$ and assumed spherical symmetry of the stationary distribution. By comparing this with the definition of current in terms of the diffusion tensor and assuming

isotropy, $\mathbf{D} \approx \frac{1}{2}\mathrm{Tr}(\mathbf{D})\mathbb{1}$, we derive at the following expression for the diffusion coefficient in the relaxation time approximation, assuming a Boltzmann distribution $n_{\mathbf{k}}$:

$$D = \frac{1}{2} \sum_{\mathbf{k}\eta} |\mathbf{v}^{\eta}_{\mathbf{k}}|^2 \tau^{\eta}_{\mathbf{k}} \frac{n^{\eta}_{\mathbf{k}}}{\frac{1}{N}\sum_{\mathbf{k}\eta} n^{\eta}_{\mathbf{k}}}. \tag{23}$$

### Monte Carlo implementation of the Boltzmann transport equation

The high dimensionality inherent to the problem makes direct numerical integration of Eq.16, for instance via Runge-Kutta methods, computationally prohibitive. An alternative and more feasible strategy is to employ a Monte Carlo approach. This method is based on a stochastic interpretation of Eq.16[11–13], where the evolution of the exciton distribution is expressed in terms of individual quasi-particle trajectories. Each exciton is characterized by the triplet $(\eta, \mathbf{r}, \mathbf{k})$, denoting its band index, position, and center of mass momentum, respectively. Between scattering events, excitons propagate as free particles with the group velocity $\mathbf{v}^{\eta}_{\mathbf{k}}$. After a time interval $\delta t$, it reaches a new position $\mathbf{r}_f = \mathbf{r} + \mathbf{v}^{\eta}\mathbf{k}\delta t$, at which point it undergoes a scattering event to a final state $(\xi, \mathbf{r}_f, \mathbf{p})$, sampled according to the transition probability $\tilde{W}^{\eta\xi}_{\mathbf{kp}}$. The algorithm is structured in two main stages: a collision step followed by an advancement step. Each particle is initialized in a specific state $(\eta, \mathbf{r}, \mathbf{k})$. In the collision step, two random numbers $n_1, n_2 \in [0, 1)$ are drawn. The first, $n_1$, is used to select the final scattering state $\xi, \mathbf{p}$ from the conditional probability distribution $\tilde{W}^{\eta\xi}_{\mathbf{kp}}$, where the initial state is fixed. The second, $n_2$, determines the time of flight based on the assumption that the survival probability (i.e., the probability that no scattering has occurred up to time $t$) decays exponentially as $\exp\left(-\int_0^t \tilde{W}^{\eta\xi}_{\mathbf{kp}}, dt'\right)$. This leads to the expression $t_f = -\ln(1 - n_2)/\tilde{W}^{\eta\xi}_{\mathbf{kp}}$ for the time interval between collisions. Following this, the advancement step updates the particle's position according to its velocity and computed flight time: $(\eta, \mathbf{r}, \mathbf{k}, t_i) \to (\xi, \mathbf{r} + \mathbf{v}^{\eta}_{\mathbf{k}}(t_f - t_i), \mathbf{p})$. By aggregating the states of all particles at a given time $t$, one can reconstruct the evolving distribution function.

### Integral evaluation of scattering tensor and self-consistent approach

In Eq. 16, the tensor $\tilde{W}^{\eta\xi}_{\mathbf{kp}}$ describes the probability of phonon-mediated scattering events between states, and from this, the dephasing rate $\Gamma^{\eta}_{\mathbf{k}}$ can be expressed as

$$\Gamma^{\eta}_{\mathbf{k}} = \frac{\hbar}{2}\sum_{\mathbf{p},\xi} \tilde{W}^{\eta\xi}_{\mathbf{kp}} = \pi \sum_{j,\mathbf{g},\mathbf{p},\xi,\pm} \left|D^{\eta\xi j}_{\mathbf{g}}(\mathbf{k},\mathbf{p})\right|^2 \left(\frac{1}{2} \pm \frac{1}{2} + n^{j}_{\mathbf{p}-\mathbf{k}+\mathbf{g}}\right) \mathcal{L}_{\Gamma^{\eta}_{\mathbf{k}}+\Gamma^{\xi}_{\mathbf{p}}}\left(E^{\xi}_{\mathbf{p}} - E^{\eta}_{\mathbf{k}} \pm \Omega_{\mathbf{p}-\mathbf{k}+\mathbf{g}}\right), \tag{24}$$

where $j$ denotes the phonon mode, and the $\pm$ corresponds to phonon emission and absorption, respectively. This expression arises from including collisional broadening due to higher-order (third-order) interaction terms[14,15]. However, it is well-known that this self-consistent formulation often leads to an overestimation of the dephasing rate[16], producing unphysical results. This discrepancy reflects the fact that the Lorentzian profile used in $\mathcal{L}$ spreads spectral weight far beyond the energy-conserving region, violating the sharp resonance condition implied by energy conservation. Consequently, contributions from energetically distant states are artificially amplified, which can lead to significantly inflated effective temperatures. To mitigate this issue while retaining a physically meaningful broadening mechanism, we replace the Lorentzian function with a generalized normal distribution. This form preserves the peak structure around the energy-conserving condition $\delta(E^{\xi}_{\mathbf{p}} - E^{\eta}_{\mathbf{k}} \pm \Omega_{\mathbf{p}-\mathbf{k}+\mathbf{g}})$, but suppresses contributions from off-resonant states more effectively. We solve Eq.24 self-consistently using this modified broadening scheme in order to compute the scattering tensors used in our simulations.

### Anomalous diffusion regime

As shown in the main text for a twist angle of 3° and temperatures below 60 K, we observe an anomalous regime of enhanced exciton diffusion caused by a non-Boltzmann distribution of excitons due to the emergence

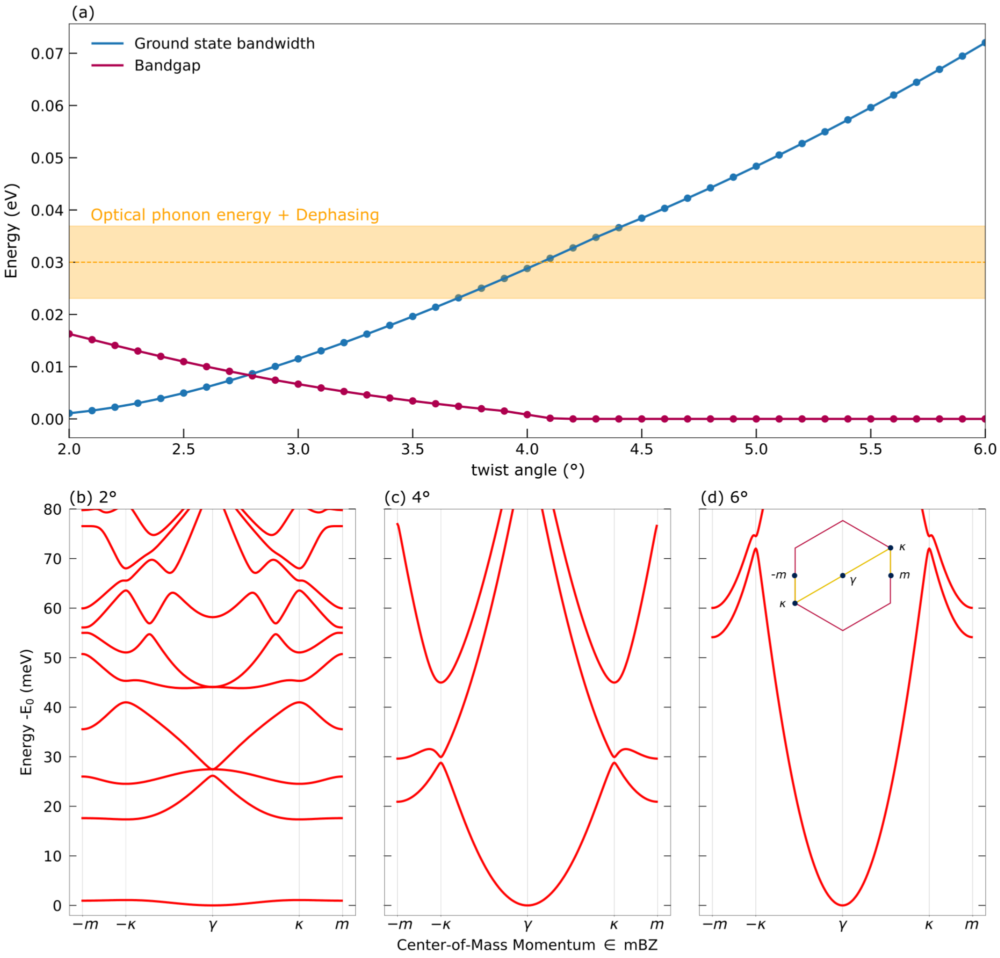


Fig. S2. (a) Twist angle dependence of the moiré exciton ground state bandwidth (blue line) and bandgap between the ground and first excited state (purple line with circles). The orange dashed line indicates the optical phonon energy, and the shaded region highlights the resonance broadening due to dephasing at temperatures around 70 K. The intersection between the orange region and the blue curve marks the upper bound, where the bottleneck is suppressed. We predict the anomalous diffusion region to extend up to approximately 3.7°, as the bandgap between the ground and first excited states (purple line) becomes considerably small allowing relaxation via acoustic phonons. (b)–(d) Exciton band structures for twist angles of 2°, 4°, and 6°, respectively.

of a pronounced phonon bottleneck effect. Note that below about 2° the anomalous exciton diffusion vanishes because the lowest moiré exciton bands become nearly completely flat (Fig. S2(b)), effectively reducing the group velocity to zero. In this regime of flatness, the Boltzmann transport equation approach is no longer adequate to describe exciton dynamics and diffusion, since excitons are localized and diffusion is strongly suppressed due to the vanishing group velocity. For twist angles between 3° and 4°, we observe a gradual onset of the anomalous diffusion regime. This behavior arises from the interplay between the moiré exciton ground-state bandwidth and the bandgap separating the ground and first excited states. Specifically, when the bandgap remains sufficiently large to suppress acoustic phonon scattering, and the bandwidth of the ground-state band is smaller than the optical phonon energy, a phonon bottleneck emerges. This bottleneck causes a non-Boltzmann population that accumulates near the band minima of the excited state, resulting in an enhanced exciton diffusion. This is illustrated in Fig. S2(a), which shows the twist-angle dependence of the moiré exciton ground-state bandwidth (blue line) and the bandgap between the ground and first excited states (purple line with circles). The orange dashed line indicates the optical phonon energy, while the shaded region highlights the resonance broadening due to dephasing at temperatures around 70 K. The intersection between the lower bound of the orange region and the blue curve marks the upper limit, beyond which

the bottleneck is suppressed. Based on this, we predict that the anomalous diffusion region extends up to approximately 3.7°, as the bandgap between the ground and first excited states becomes small enough to allow relaxation via acoustic phonons.

---

* giuseppe.meneghini@physik.uni-marburg.de

[1] Simon Ovesen, Samuel Brem, Christopher Linderälv, Mikael Kuisma, Tobias Korn, Paul Erhart, Malte Selig, and Ermin Malic, "Interlayer exciton dynamics in van der waals heterostructures," Communications Physics **2**, 1–8 (2019).

[2] Samuel Brem, Kai-Qiang Lin, Roland Gillen, Jonas M Bauer, Janina Maultzsch, John M Lupton, and Ermin Malic, "Hybridized intervalley moiré excitons and flat bands in twisted wse 2 bilayers," Nanoscale **12**, 11088–11094 (2020).

[3] Jin Z, Li X, Mullen J T, and Kim K W, "Intrinsic transport properties of electrons and holes in monolayer transition-metal dichalcogenides," Phys. Rev. B **90**, 045422 (2014).

[4] Xiaobo Lu, Xiaoqin Li, and Li Yang, "Modulated interlayer exciton properties in a two-dimensional moiré crystal," Physical Review B **100**, 155416 (2019).

[5] Samuel Brem, Christopher Linderälv, Paul Erhart, and Ermin Malic, "Tunable phases of moiré excitons in van der waals heterostructures," Nano letters **20**, 8534–8540 (2020).

[6] Ortwin Hess and Tilmann Kuhn, "Maxwell-bloch equations for spatially inhomogeneous semiconductor lasers. i. theoretical formulation," Physical Review A **54**, 3347 (1996).

[7] Raul Perea-Causin, Samuel Brem, Roberto Rosati, Roland Jago, Marvin Kulig, Jonas D Ziegler, Jonas Zipfel, Alexey Chernikov, and Ermin Malic, "Exciton propagation and halo formation in two-dimensional materials," Nano letters **19**, 7317–7323 (2019).

[8] Roberto Rosati, Robert Schmidt, Samuel Brem, Raül Perea-Causín, Iris Niehues, Johannes Kern, Johann A Preuß, Robert Schneider, Steffen Michaelis de Vasconcellos, Rudolf Bratschitsch, *et al.*, "Dark exciton anti-funneling in atomically thin semiconductors," Nature Communications **12**, 7221 (2021).

[9] Roberto Rosati, Raül Perea-Causín, Samuel Brem, and Ermin Malic, "Negative effective excitonic diffusion in monolayer transition metal dichalcogenides," Nanoscale **12**, 356–363 (2020).

[10] Jiaqi He, Dawei He, Yongsheng Wang, Qiannan Cui, Frank Ceballos, and Hui Zhao, "Spatiotemporal dynamics of excitons in monolayer and bulk ws2," Nanoscale **7**, 9526–9531 (2015).

[11] Jean-Philippe M Péraud, Colin D Landon, and Nicolas G Hadjiconstantinou, "Monte carlo methods for solving the boltzmann transport equation," Annual Review of Heat Transfer **17** (2014).

[12] Tilmann Kuhn and Fausto Rossi, "Monte carlo simulation of ultrafast processes in photoexcited semiconductors: Coherent and incoherent dynamics," Physical Review B **46**, 7496 (1992).

[13] Carlo Jacoboni and Lino Reggiani, "The monte carlo method for the solution of charge transport in semiconductors with applications to covalent materials," Reviews of modern Physics **55**, 645 (1983).

[14] J Schilp, T Kuhn, and G Mahler, "Electron-phonon quantum kinetics in pulse-excited semiconductors: Memory and renormalization effects," Physical Review B **50**, 5435 (1994).

[15] J Schilp, T Kuhn, and G Mahler, "Quantum kinetics of the coupled carrier-phonon system in photoexcited semiconductors," physica status solidi (b) **188**, 417–424 (1995).

[16] Fausto Rossi and Tilmann Kuhn, "Theory of ultrafast phenomena in photoexcited semiconductors," Reviews of Modern Physics **74**, 895 (2002).
